# Hemiplegia after Scarlet Fever

**Published:** 1908-07-25

**Authors:** 


### HEMIPLEGIA AFTER SCARLET FEVER.

Gross lesions of the nervous system are not very common after any of the specific fevers except diphtheria, but now and then a case is seen after one of the others. Hemiplegia or paraplegia from vascular thrombosis and softening in the brain or in the spinal cord is not unknown after typhoid fever, and similar lesions are possible after scarlatina.

A man of about 20, who had hitherto been in perfect health, whose family history was good, and who had not suffered from syphilis, was taken ill with severe scarlet fever. On the fifth day all his symptoms began to abate, and the disease seemed to be following a normal course, when suddenly at noon of the seventh day the patient was, without warning, seized with a grave apoplectic attack, in which he lay completely comatose with paralysis of the left leg, left arm, and the left side of his face. This hemiplegia was at first accompanied by transient twitchings and contractions in the affected parts. For a while the prognosis seemed very bad; but the coma disappeared on the third day, and the temperature dropped nearly to normal, though the hemiplegia, together with incontinence of urine and fæces, persisted. There was complete loss of all power on the left side, except in the forehead and eyelids; and there was left hemianæsthesia as regards cutaneous sensibility, but not as regards the other kinds of sensation. The paralysis slowly mended, the hemianæsthesia disappearing first.

Eight months from the beginning of the trouble the left arm was in a state of contracture in a semi-flexed position. The forearm was bent to a right angle with the upper arm at the elbow; the hand was fully flexed at the wrist, and the fingers were all firmly bent in towards the palm. No voluntary movement was possible in the fingers nor at the wrist, elbow, or shoulder; the most that the patient could manage was to abduct slightly the whole arm from the trunk. The left leg was in a position of extension; no voluntary movements could be made in the toes or at the ankle; the knee could be only slightly flexed, but movements at the hip joint were pretty good. Lying in bed, the leg could be strongly raised from the mattress, but walking was a matter of great difficulty and stumbling. There was still paresis of the left side of the face, evident both when the features were at rest and when they were voluntarily put into action.

All tendon reflexes, normal or slightly increased upon the right side, were very exaggerated on the left, both in the arm and in the leg. The left plantar reflex was extensor, the right flexor. The cremasteric reflex was diminished on the left side.

Sensibility to touch and to a pin-prick was retained, but there were delay and uncertainty in locating sensations properly on the left side, especially in the arm and leg; alterations in the positions of the paralysed limbs were also ill-perceived, so that there was some impairment of the muscle sense. There was no hemianopsia nor other disturbance of vision; hearing, taste, and smell were natural. There were no zones of hyperæsthesia, no pain on pressure over the vertex, the vertebral column, the epigastric angle, or the iliac fossæ, nor any anæsthesia of the pharynx suggestive of neurosis. The lungs, heart, abdominal organs, and urine were all natural.

In yet another case, also that of a young man of good family and personal history, there had been an ordinary attack of scarlet fever, and on the morning of the sixth day, suddenly and without warning, coma supervened. The coma was of less duration, however, than in the former patient, lasting a few hours only, and the left hemiplegia was partial only, the face almost escaping, and there being but slight anæsthesia. About the eighth day the temperature rose again, and there was intense headache, somnolence, pains, and involuntary starts in the left arm and leg, and incontinence of urine and fæces.

A few weeks later, however, movement and sensibility had returned in the left leg to such an extent that walking was possible without much difficulty. The arm was still much affected. Eight months from the onset the left arm could perform all the ordinary movements, and at first sight showed no sign of paralysis. Its action, however, was slower and less forcible than before. The left leg was thoroughly useful, but the patient complained of its seeming to be stiff. The face was quite normal. The tendon reflexes in the left arm and leg were still brisker than those in the right, but upon the whole the patient could be regarded as quite recovered. He presented none of the stigmata of hysteria.

The two cases are therefore very much alike except in degree. In the one case the trouble resolved in the course of a few months; in the other, where hemianæsthesia was a marked feature, recovery was very imperfect. There could be little doubt about the lesion being organic in each case; the extensor plantar reflex (Babinski's sign) was by itself enough to prove this.

The only remaining question is as to the nature of the lesion and its site. A capsular hæmorrhage resulting from weakness of the vessel walls seems unlikely; the suggestion might be entertained if there had been evidence of a tendency to hæmorrhage elsewhere, but there was neither epistaxis, purpura, hæmatemesis, nor hæmaturia. Embolism seemed unlikely, for the heart sounds were quite normal.

It is more probable that the lesion was a softening due to an endarteritis with or without thrombosis. In favour of this view is the record, by Alexeieff, of an autopsy in a similar case, in which there was softening of almost the whole of the lenticular nucleus and of the posterior part of the internal capsule, and a thrombosis of the middle cerebral artery. Moreover, a precisely analogous lesion has more than once been found in cases of typhoid fever.

As to the site of the thrombosis and softening, it may have been either capsular or cortical; but there are certain points favouring the latter rather than the former; in the first place the twitchings and starts that occurred in the paralysed limbs shortly after the hemiplegia developed suggest an irritation of the pyramidal cells rather than of the fibres below them; the contractures in the first case point in the same direction; and the hemi-anæsthesia was restricted to impairment of general sensibility without any affection of sight.

